# Histological Lesions and Cellular Response in the Skin of Alpine Chamois (*Rupicapra r. rupicapra*) Spontaneously Affected by Sarcoptic Mange

**DOI:** 10.1155/2016/3575468

**Published:** 2016-06-15

**Authors:** Claudia Salvadori, Guido Rocchigiani, Camilla Lazzarotti, Nicoletta Formenti, Tiziana Trogu, Paolo Lanfranchi, Claudia Zanardello, Carlo Citterio, Alessandro Poli

**Affiliations:** ^1^Department of Veterinary Sciences, University of Pisa, Viale delle Piagge 2, 56124 Pisa, Italy; ^2^Department of Veterinary Sciences and Public Health, University of Milan, Via Celoria 10, 20132 Milano, Italy; ^3^Istituto Zooprofilattico Sperimentale delle Venezie, SCS3 Diagnostica Specialistica e Istopatologia, Viale dell'Università 10, 35020 Legnaro, Italy; ^4^Istituto Zooprofilattico Sperimentale delle Venezie, SCT2 Belluno, Via M. Cappellari 44, 32100 Belluno, Italy

## Abstract

Population dynamics of chamois (genus* Rupicapra*, subfamily Caprinae) can be influenced by infectious diseases epizootics, of which sarcoptic mange is probably the most severe in the Alpine chamois (*Rupicapra rupicapra rupicapra*). In this study, skin lesions and cellular inflammatory infiltrates were characterized in 44 Alpine chamois affected by sarcoptic mange. Dermal cellular responses were evaluated in comparison with chamois affected by trombiculosis and controls. In both sarcoptic mange and trombiculosis, a significantly increase of eosinophils, mast cells, T and B lymphocytes, and macrophages was detected. Moreover, in sarcoptic mange significant higher numbers of T lymphocytes and macrophages compared to trombiculosis were observed. Lesions in sarcoptic mange were classified in three grades, according to crusts thickness, correlated with mite counts. Grade 3 represented the most severe form with crust thickness more than 3.5 mm, high number of mites, and severe parakeratosis with diffuse bacteria. Evidence of immediate and delayed hypersensitivity was detected in all three forms associated with diffuse severe epidermal hyperplasia. In grade 3, a significant increase of B lymphocytes was evident compared to grades 1 and 2, while eosinophil counts were significantly higher than in grade 1, but lower than in grade 2 lesions. An involvement of nonprotective Th2 immune response could in part account for severe lesions of grade 3.

## 1. Introduction

Sarcoptic mange is a worldwide, highly contagious, burrowing mite infection in the skin of humans and animals, caused by* Sarcoptes scabiei* [1, 2]. Sarcoptic mange is responsible for epizootic disease in wildlife populations [2] and represents one of the most severe infections in Alpine chamois (*Rupicapra rupicapra rupicapra*, Linnaeus 1758). A study on chamois populations of Italian Alps [3] showed that the first impact of mange on naive host populations can be dramatic, with mortality rates of over 80%. After this mortality peak, populations tend to recover and the following peaks of the disease, generally 10–15 years after the first one, have a far less severe impact but could seriously affect fragmented populations with limited exchange with each other [2, 3]. Mortality in Alpine chamois has been used as a proxy of the sensitivity of different classes to the disease: no significant differences have been observed in mange-related mortality by gender, while an evaluation according to the age is still difficult due to the definitely lower probability of detection of smaller individuals (young chamois) in the field compared to the adult chamois in the framework of passive surveillance [4]. On the other hand, mange cases show seasonality, being more frequent in late winter and spring [4, 5].

Sarcoptic mange skin lesions in wild and domestic animals are generally a combination of crusts and alopecia with dermatitis, orthokeratosis, and epidermal hyperplasia. However, severity and distribution of the lesions as well as the disease outcome vary among different host species and among individuals of the same species, probably due to different level of immune response and/or different clinical stages [1, 2]. Affected chamois showed clinical symptoms like restlessness, itch, and crusted alopecic skin, mainly involving head, neck, abdomen, and limbs [2, 3]. Histologically, orthokeratosis and parakeratosis, epidermal hyperplasia, and formation of crusts were observed. Vasodilation with perivascular and interstitial inflammatory infiltrates composed of lymphocytes, macrophages, eosinophils, plasma cells, neutrophils, and mast cells was detected suggesting a delayed hypersensitivity response [6, 7]. A study on* R. pyrenaica* suggested that sarcoptic mange infection elicits also a humoral immune response with increase of IgG levels in infected chamois associated with higher values of total proteins and gamma-globulin [8]. However, as in human scabies, it is uncertain whether increase of antibodies level can be specific or related to associated secondary bacterial infection [9]. In European wild ruminants, detailed histological features of sarcoptic mange have been described in Spanish ibex (*Capra pyrenaica hispanica*) [10], but* Sarcoptes* infection has been described also in ibex (*Capra ibex*) [3], Cantabrian chamois [7], Barbary sheep (*Ammotragus lervia*) [11], roe deer (*Capreolus capreolus*), and red deer (*Cervus elaphus*) [12]. An immunohistochemical study on formalin fixed skin specimens of normal and sarcoptic mange-infected chamois showed a progressive loss of cytokeratins in the epidermis and follicular epithelium in the orthokeratotic and parakeratotic form but failed to demonstrate reactivity of immune cells with a panel of anti-human antibodies [6].

In order to clarify the pathogenesis of dermal changes in Alpine chamois sarcoptic mange, the aims of this study were (1) to provide detailed histological features description of* Sarcoptes scabiei* var.* rupicaprae* infection in this species and (2) to evaluate dermal immune response in different forms of sarcoptic lesions. To evaluate and interpret inflammatory cells composition of mange cutaneous infiltrates, a group of chamois belonging to different districts and affected by trombiculosis was also evaluated.

## 2. Materials and Methods

### 2.1. Animals

Skin samples were collected from 75 Alpine chamois (*R. r. rupicapra*) ageing between one and thirteen years and culled during hunting seasons from 2013 to 2015. Three groups of chamois were selected: group 1 (*n* = 44) from Belluno province (46°53′N, 12°14′E) a mountainous area with altitudes ranging from less than 500 metres to over 3000 metres above the sea level (a.s.l.) affected by sarcoptic mange; group 2 (*n* = 18) from Lecchesi Alps and Pre-Alps hunting districts (45°59′N, 9°32′E), ranging from 300 to >2,000 m a.s.l. and from Val d'Ossola (46°07′N, 8°17′E) altitude ranging from 700 to 2,400 m a.s.l. affected by trombiculosis; group 3 consisting of 13 healthy chamois from all three previous districts. In each animal a 2 × 2 cm square skin sample was collected from affected skin or from lateral aspect of thigh in control animals. Samples were placed in a freshly prepared zinc salts fixative (ZSF) for 24–48 hours. All samples were processed routinely for paraffin embedding. Serial 5 *μ*m sections from all specimen were stained for haematoxylin and eosin (HE) and Toluidine Blue (TB) and mounted on treated glass slides (SuperFrost Plus, Menzel-Glaser, Germany), respectively.

### 2.2. Histopathology

Histologic examination was performed on all 75 chamois. Characteristics of mange lesions were appreciated by describing crusting (thickness in mm), alopecia (percentage of hair follicle containing hairs in histologic skin sections), mites (counts at 10x HPF in three randomly selected fields), and dermal inflammatory cells infiltration using the scoring system proposed by Nimmervoll et al. [1] modified as proposed in Table 1. Data about the presence of bacteria in the crusts, orthokeratosis, parakeratosis, epidermal hyperplasia, spongiotic oedema, hypergranulosis, epidermal erosions, presence of pustule, sebaceous hyperplasia, and fibrosis were also recorded when observed.

### 2.3. Immunohistochemistry

Sections were dewaxed in xylene and rehydrated through graded alcohols prior to quenching endogenous peroxidase activity with H_2_O_2_ 3% in methanol for 20 minutes. Immunohistochemical labelling was performed manually with the Sequenza slide rack and Coverplate system (Shandon, Runcorn, UK). Nonspecific antigen binding was blocked by incubation with 25% normal goat serum (code X0907, Dako UK Ltd., Ely, UK). Monoclonal anti-human CD3 (1 : 100, clone F7.2.38, Dako, UK Ltd., Ely, UK), CD79*α* (1 : 50, clone HM57, Dako, UK Ltd., Ely, UK), and CD68 (1 : 50, clone EBM 11, Dako, UK Ltd., Ely, UK) antibodies were applied to serial sections and incubated overnight at 4°C. Antibody binding was detected by the EnVision Plus System-HRP (DAB) (code K4007, Dako UK Ltd., Ely, UK) as indicated by manufacturer's instructions and slides were counterstained with haematoxylin. Substitution of the primary antibody with unrelated matched primary antibody was used to provide a negative control. Serial sections of chamois lymph node were used as positive control.

### 2.4. Cell Counting and Statistical Analysis

Four bright field images for each skin sample were acquired at ×20 magnification with a Leica Microsystem DFC490 digital camera mounted on Leica DMR microscope. Number of eosinophils, mast cells, CD3, CD79*α*, and CD68-positive cells were counted on eight 10,000 *μ*m^2^ random fields of each skin sample using a semiautomatic analysis system (LASV 4.3, Leica, Germany). Eosinophils were counted on HE sections and mast cells were counted on TB stained slides.

Statistical analysis was performed using the statistical package SPSS Advanced Statistics 21.0 (SPSS Inc., Chicago, IL, USA). ANOVA test was used to compare the composition of cell infiltrates detected in the skin of animals with sarcoptic mange, trombiculosis, and controls. Post hoc analysis was made by Bonferroni Test. Statistical significance was based on a 5% (0.05) significance level. The statistical correlation between mite counts and crust thickness was performed using the Pearson correlation test.

## 3. Results

### 3.1. Histopathologic Findings

#### 3.1.1. Sarcoptic Mange

**Figure 1:**
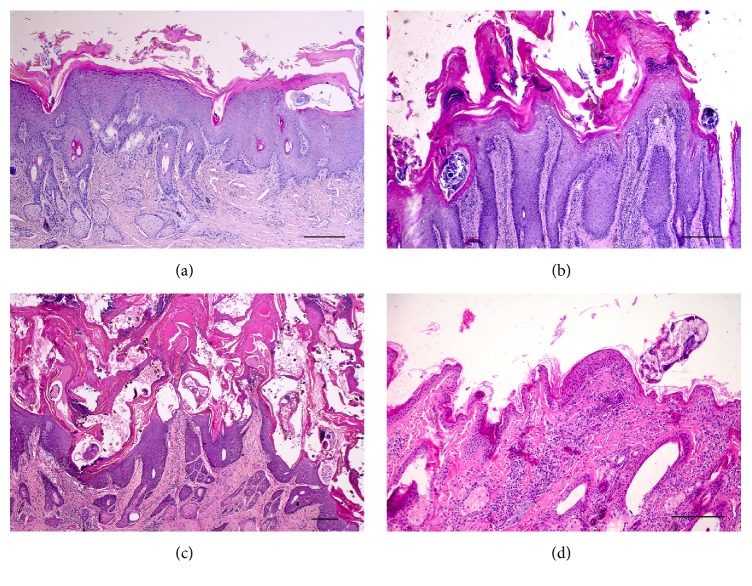
Histology of skin in sarcoptic (a: grade 1; b: grade 2; c: grade 3) and trombiculosis (d) affected chamois. Severe epidermal hyperplasia is evident in all grades of sarcoptic mange lesions with females within epidermal layers. Crusting with marked parakeratosis is progressively severe from grade 1 to grade 3. In grade 3 (c), crusts are associated with serum lakes, extravasated erythrocytes, and bacteria. Diffuse inflammatory infiltrates are evident in the dermis. Focal, mild epidermal hyperplasia is evident in trombiculosis (d) with a mite on the surface of the keratin layer and no crusts. Focal inflammatory infiltrates are evident in the dermis. Haematoxylin Eosin; bar = 100 *μ*m.

In all sarcoptic mange cases, variable crusting was observed (Figure 1). Crusts thickness, measured on histologic sections, ranged from mild to severe. Mild crusting (grade 1) was observed in 7/44 chamois (15.9%), while moderate crusting (grade 2) and severe crusting (grade 3) were detected in 17/44 (38.6%) and 20/44 animals (45.5%), respectively. Crusts, generally, containing a large number of mites and multifocal to diffuse colonies of bacteria, showed severe parakeratosis and moderate orthokeratosis, occasionally serum lakes, and extravasated erythrocytes. Generally, crust thickness increased with mite counts within the crusts (*r* = 0.677, *p* = 0.000) and cases with severe crusting showed higher presence of bacteria. Alopecia was commonly seen in chamois with severe crusting; however, correlation between crusting and alopecia was not significant.

In all mangy forms (all grades of crusting severity), moderate to very severe epidermal hyperplasia was observed with conspicuous, diffuse rete ridge formation. Diffuse spongiotic oedema of the epidermal layer was observed in 9 animals (9/44, 20.5%) (Figures 1(b) and 1(c)) and mild hypergranulosis was observed in 18 chamois (18/44, 41.0%). Focally extensive to multifocal epidermal erosions were observed in 13 (13/44, 29.5%) animals. Focal or multifocal pustules with neutrophils and/or eosinophils were present in 10 (10/44, 22.7%) animals and generally involved cases with focal to multifocal epidermal erosions. Moderate sebaceous gland hyperplasia was observed in 31 (31/44, 70.5%) cases but was not correlated with crusting severity. Varying degrees of inflammatory cells infiltration were present, mainly in the superficial and also in the deep dermis with a diffuse and less perivascular distribution. Eosinophils, lymphocytes, plasma cells, and macrophages were detected with few numbers of mast cells. Dermal fibrosis was frequently observed and was more pronounced in grade 3 lesions.

#### 3.1.2. Trombiculosis

Trombiculid mites were localized on the surface of the epidermis, over the keratin layer (Figure 1(d)). Generally, a low number of mites (1-2 mites at 10x HPF) were detected with moderate histological lesions. Moderate parakeratosis with slight crusts (1.3 mm of thickness) and multifocal erosions was observed only in a chamois with high number of mites (3 mites/HPF), but generally no crusts or orthokeratosis was evident in chamois with trombiculid mites. No epidermal hyperplasia and alopecia were observed in all chamois with trombiculosis. In the superficial dermis, an eosinophilic stylostome, with a variable angle with skin surface, was associated with mites and surrounded by a focal granulomatous reaction, with macrophages, lymphocytes, and eosinophils. Inflammatory cells were also slightly diffuse in the surrounding superficial dermis.

#### 3.1.3. Control Chamois

In nonaffected chamois, no epidermal hyperplasia, crusts, orthokeratosis, or alopecia was detected. In the superficial dermis, mild infiltration of lymphocytes, macrophages, and rare, single eosinophils were detected (not shown).

#### 3.1.4. Inflammatory Cells

**Figure 2:**
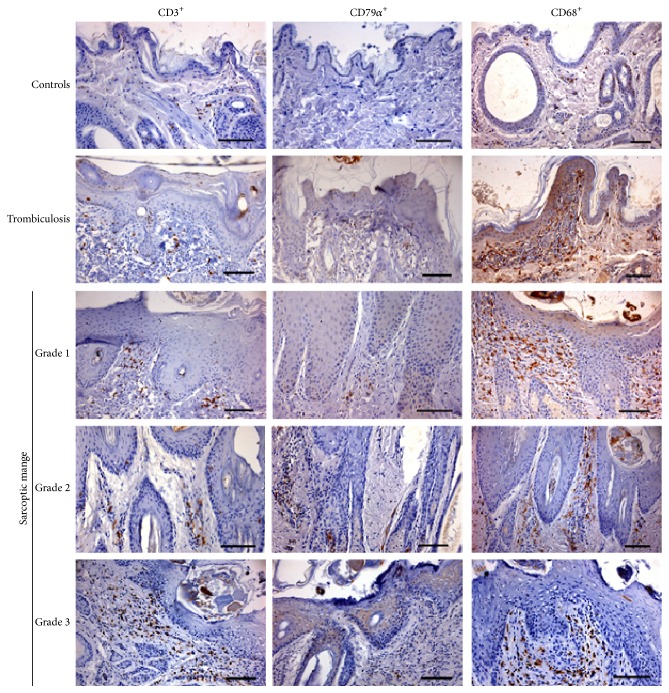
Immunohistochemical staining of skin of control, trombiculosis, and mange affected chamois. Scanty T lymphocytes (CD3^+^ cells) and macrophages (CD68^+^ cells) and very rare B lymphocytes (CD79*α* ^+^ cells) are evident in control chamois. Focal inflammatory infiltration with a prevalence of macrophages is evident in trombiculosis-affected chamois. A prevalence of macrophages and T lymphocytes is evident in all three groups of sarcoptic mange affected chamois. DAB chromogen and haematoxylin counterstain. Bar = 100 *μ*m.

**Figure 3:**
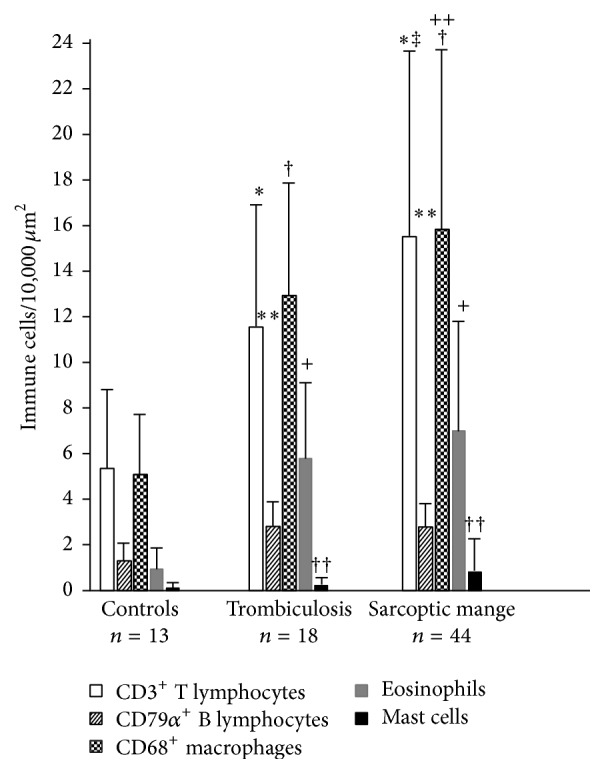
Histogram of number of immune cells in 10,000 *μ*m^2^ of skin in control, trombiculosis, and mange affected chamois. Significant differences among CD3^+^ T lymphocyte (*∗*), CD79*α* ^+^ B lymphocyte (*∗∗*), CD68^+^ macrophages (†), eosinophils (+), and mast cells (††) with controls. Significant differences between CD3^+^ T lymphocyte (‡) and CD68^+^ macrophages (++) in chamois with sarcoptic mange and subjects with trombiculosis.

T and B lymphocytes and macrophages were correctly identified in chamois skin with immunohistochemistry (Figure 2). Counts of the different cell subsets present in the inflammatory infiltrates are presented in Table 2 and Figure 3. In the skin of parasitized chamois, with both sarcoptic mites and trombiculid, there was a significant increase of dermal inflammatory cells such as T and B lymphocytes (*p* = 0.000 and *p* = 0.000, resp.), macrophages (*p* = 0.000), eosinophils (*p* = 0.000), and mast cells (*p* = 0.000), when compared with control chamois. Moreover, there was a significantly higher number of T lymphocytes and macrophages in chamois with sarcoptic mange compared to chamois with trombiculosis (*p* = 0.000 and *p* = 0.001, resp.). Finally, in sarcoptic mange cases there was higher number of eosinophils (6.5* versus* 5.5 eosinophils/0.01 mm^2^) compared to animal with trombiculosis, although this difference was not statistically significant.

#### 3.1.5. Sarcoptic Mange

**Figure 4:**
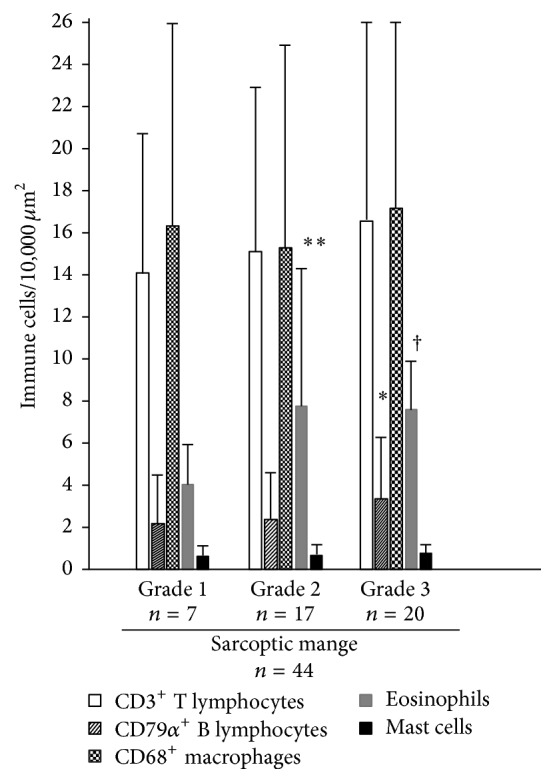
Histogram of number of immune cells in 10,000 *μ*m^2^ of skin in mange affected chamois, regarding different grading of sarcoptic mange lesions. Significant differences among CD79*α* ^+^ B lymphocyte (*∗*) in chamois with grade 3 and subjects with grades 1 and 2; significant differences among eosinophils (*∗∗*) in chamois with grade 2 and animals with grades 1 and 3; significant differences between eosinophils (†) in chamois with grade 3 and grade 1.

Epidermal exocytosis were observed mainly for T lymphocytes and CD68-positive cells. The last cells were interpreted as exocytosis of macrophages or Langerhans cells. However, no inflammatory cells within epidermal layer were counted in this study. T lymphocytes and macrophages were the prevalent cells in inflammatory infiltrates of mangy chamois with higher number of CD68-positive cells in 0.01 mm^2^. These cells were present in dermal infiltration with comparable levels in different crusting severity. Counts of the different cell subsets present in the inflammatory infiltrates are presented in Table 2 and Figure 4. Indeed, T lymphocytes lightly increased from grade 1 to grade 3 but differences were not significant. Conversely, B lymphocytes were significantly more numerous in animals with grade 3 lesions than in animals with grades 1 and 2 lesions (*p* = 0.05). Significantly a higher number of eosinophils were evident in grade 2 (7.8 eosinophils/0.01 mm^2^) compared to grade 3 lesions (6.2 eosinophils/0.01 mm^2^; *p* = 0.05) and grade 1 form (4.0 eosinophils/0.01 mm^2^; *p* = 0.001). Moreover, a significant higher number of eosinophils were evident in grade 3 compared to grade 1 lesions (*p* = 0.05). Mast cells were present with comparable numbers in all three groups of sarcoptic lesions.

## 4. Discussion

In this study, histopathological features and inflammatory infiltrates in Alpine chamois skin affected by sarcoptic mange were characterized. ZS fixed specimens allowed a complete evaluation of inflammatory cells. With a comparison purpose, trombiculosis skin lesions were also characterized. As expected, an increase of dermal inflammatory cells such as eosinophils, T and B lymphocytes, and macrophages was observed in the skin of parasitized chamois with both sarcoptic mites and trombiculid mite larvae. Generally, a lower number of mites and less severe lesions were observed in trombiculosis than sarcoptic mange. Indeed, in trombiculosis, focal lesions with one or two parasites were associated with moderate inflammatory infiltrates with granulomatous features. However, being trombiculid mites on the surface of the epidermis (so they could be removed during fixation and embedding) and stylostomes not always detectable, the impact of trombiculosis could be histologically underestimated.

Lesions due to sarcoptic mange were consistent with other studies in chamois and other species [1, 2, 6, 7, 13–15]. Crusts, parakeratosis, severe epidermal hyperplasia, abundance of mites, bacterial colonies, mixed dermal infiltration, sebaceous gland hyperplasia, and fibrosis were the most frequent histopathological features in affected chamois. Inflammatory infiltrates had a diffuse and perivascular distribution and were characterized by a prevalence of macrophages and lymphocytes, with eosinophils and rare mast cells. Epidermal hyperplasia was a common feature in all three grades of crusting severity. Indeed, epidermal disruption by burrowing female mites is known to initiate keratinocyte hyperproliferation also in early stages of sarcoptic infection [16]. Subsequently, in severe cases, activated CD8+ lymphocytes may induce dysregulated keratinocyte apoptosis contributing to the elicitation and progress of epidermal hyperproliferation [9]. In sarcoptic chamois, the number of T lymphocytes and macrophages in the dermis was significantly higher than in chamois with trombiculid mites and controls animals, consistent with a dermal inflammatory response. Indeed, the mite secretes compounds that dissolve the stratum corneum of the epidermis, burrowing through this dead cell layer until it reaches and consume live cells of the underlying layers of the epidermis. In all stages, mite and its products such as secretory products, exuvia, faeces, and eggs present antigens towards which hosts produce hypersensitivity reaction [14, 15]. Infiltration of Langerhans cells was observed in the epidermis, and it is assumed that epidermal Langerhans cells internalize sarcoptic antigen, migrate to draining regional lymph node, and stimulate T cells [17].

Inflammatory infiltrates varied between grades of severity. Grade 3 lesions were characterized by an increase of T and B lymphocytes, macrophages, and eosinophils compared to grade 1. However, only the increased numbers of B lymphocytes and eosinophils of grade 3 compared to grade 1 were significant. Moreover, in grade 3 lesions eosinophils were significantly lower than grade 2. In all stages, both type I (immediate) and type IV (delayed) hypersensitivity cells are evident, eosinophils and mast cells and lymphocytes and macrophages, respectively. In several cases we observed also signs of cytotoxicity (diffuse epidermal spongiosis). Involvement of immediate and delayed hypersensitivity immune response had been described also in other wild and domestic animals [2] as well as in humans [17]. In all mange stages, we detected a moderate number of B lymphocytes and plasma cells, higher than in controls and similar to trombiculosis-affected chamois. This feature could be indicative of hosts that have been exposed fairly recently to* Sarcoptes*. Indeed, in other free living animals such as foxes or wombats, rarely plasma cells or B lymphocytes were detected [1, 13], because some tolerance to infection with* S. scabiei* could occur with a reduction of the humoral response. However, during the immune response to arthropod antigens, desensitisation with exhaustion or suppression of antibody-producing could be the last event [13]. No significant differences were observed in the numbers of mast cells in the three grades forms of sarcoptic mange. Also, in experimentally infected Cantabrian chamois (*R. pyrenaica parva*) no mast cells hyperplasia was observed [7]. This finding differs from studies on foxes or wombats, where mast cells increased with severity of sarcoptic lesions suggesting that mast cells could not have a determining role in the pathogenesis of disease [1, 2].

According to our results, grades 1 and 2 with slight and moderate crusting and lower number of mites could be initial stages and grade 3 could be the following, generalized, severe stage. However, analogously to ordinary scabies in human beings, grade 1 and grade 2 could be suggestive of skin immune response to* S. scabiei* with adequate presence of macrophages, T lymphocytes, and eosinophils that allows a parasite control with reduction of mite numbers and limitation of lesions. Differently, grade 3 form with significant increase of B lymphocytes and decrease of eosinophils not associated with a significant increase of macrophages and T lymphocytes could be related with an involvement of nonprotective Th2 response that could be unable to control or reduce the mite burden particularly in sequential infestations [9]. However, evaluation of genetic polymorphism, in particular of MHC (major histocompatibility complex) class II, could be worthy to elucidate if animals with severe crusting really have a different genotype that account for a nonprotective immune response.

In conclusion, our study allowed us to characterize in detail the histopathological changes associated with* Sarcoptes* infection in the chamois, to classify these changes accordingly to their severity, and to define, for the first time, the cellular subsets present in the inflammatory infiltrates of lesions of different grade. The increase of B lymphocytes and eosinophils in grade 3 lesions highlights how an involvement of a nonprotective Th2 response could in part be responsible for the development of these more severe forms.

## Figures and Tables

**Table 1:** Scoring system used for classification of sarcoptic mange skin lesions determined by histologic assessment of skin sections from affected chamois.

Feature	Measure	Attributed scores			
		Absent (0)	Mild (1)	Moderate (2)	Severe (3)
Crusts	Thickness^a^	No crust	<2.5	2.5–3.5	>3.5
Alopecia	Percent of hair follicles containing hairs in histologic section	Normal hair	>55	45–55	<45
Mites	Average counts at 10x HPF^b^	0	1-2	3–6	>6
Eosinophils	Average counts at 40x HPF^c^	0-1	2–10	11–20	>20
Lymphocytes	Average counts at 40x HPF^c^	0	1–15	16–50	>50
Mast cells	Average counts at 40x HPF^c^	0	1–15	16–40	>40

^a^Thickness (mm) determined in eight randomly selected microscopic cross sections of skin. ^b^Counts of mites in eight randomly selected fields. ^c^Cell number in eight randomly selected fields. HPF: high-powered field.

**Table 2:** Subsets of inflammatory cells in skin samples from controls and trombiculid and sarcoptic mange-infected chamois.

Examined subjects	Classification of sarcoptic mange skin lesions	CD3^+^ lymphocytes/10,000 *μ*m^2^	CD79*α* ^+^ lymphocytes/10,000 *μ*m^2^	CD68^+^ macrophages/10,000 *μ*m^2^	Eosinophils/10,000 *μ*m^2^	Mast cells/10,000 *μ*m^2^
		Mean ± SD	Mean ± SD	Mean ± SD	Mean ± SD	Mean ± SD
Controls		5.4 ± 4.8	1.3 ± 1.9	5.0 ± 4.0	0.9 ± 1.7	0.1 ± 0.3
Trombiculosis		10.6 ± 7.1^*∗*^	2.9 ± 3.0^†^	13.0 ± 11.7^+^	5.5 ± 3.8^#^	0.3 ± 0.4
Sarcoptic mange	Grade 1	14.1 ± 7.5	2.3 ± 2.3	16.5 ± 10.0	4.0 ± 2.4	0.9 ± 0.7
	Grade 2	15.2 ± 7.6	2.4 ± 2.4	15.2 ± 10.1	7.8 ± 6.8^†††^	1.0 ± 0.6
	Grade 3	16.7 ± 10.3	3.3 ± 3.0^*∗∗∗*^	16.9 ± 9.2	6.2 ± 3.8^‡‡‡^	1.0 ± 0.6
	Total	15.7 ± 8.9^*∗*‡^	2.9 ± 2.8^†^	16.2 ± 9.7^+††^	6.5 ± 5.2^#^	1.0 ± 0.7^*∗∗*‡‡^

Significant differences among CD3^+^ T lymphocyte (*∗*), CD79*α* ^+^ B lymphocyte (†), CD68^+^ macrophages (+), eosinophils (#) and mast cells (*∗∗*) with controls. Significant differences between CD3^+^ T lymphocyte (‡), CD68^+^ macrophages (††) and mast cells (‡‡) in chamois with sarcoptic mange and subjects with trombiculosis. Significant differences among CD79*α* ^+^ B lymphocyte (*∗∗∗*) in chamois with grade 3 and subjects with grade 1 and 2; significant differences among eosinophils (†††) in chamois with grade 2 and animals with grade 1 and 3; significant differences between eosinophils (‡‡‡) in chamois with grade 3 and grade 1.
